# Photo-protective role of ATG5/ATG7-independent alternative autophagy in human keratinocytes

**DOI:** 10.1080/27694127.2024.2396212

**Published:** 2024-09-05

**Authors:** Tatsuya Hasegawa, Masaya Nakashima, Satoru Torii, Shinya Honda, Shigeomi Shimizu

**Affiliations:** a MIRAI Technology Institute, Shiseido Co., Ltd., Yokohama, Japan; b Department of Pathological Cell Biology, Medical Research Institute, Tokyo Medical and Dental University, Tokyo, Japan

**Keywords:** Alternative autophagy, GOMED, inflammasome, keratinocyte, mitochondria, NLRP3, skin, sunburn, UV

## Abstract

Excessive exposure to sunlight, especially to ultraviolet B (UVB), results in DNA damage and a cutaneous inflammatory reaction commonly known as sunburn, which increases skin cancer risks. UVB-induced inflammasome activation in epidermal keratinocytes mediates the cutaneous inflammatory response, but the intracellular machinery that maintains skin homeostasis by suppressing UVB-induced inflammasome activation is unclear. Here, we summarize our recent work on the protective role of alternative autophagy against UVB-induced NLRP3 (NLR family pyrin domain containing 3) inflammasome activation in human keratinocytes. We found that UVB radiation induces ATG5/ATG7-independent alternative (noncanonical) autophagy, which leads to suppression of NLRP3 inflammasome activation through the clearance of damaged mitochondria in UVB-irradiated keratinocytes. Our findings indicate that ATG5/ATG7-independent alternative autophagy, rather than conventional autophagy, may play a key role in mitigating inflammatory responses, and restoring skin homeostasis after UV radiation.

The skin, which is at the interface between our body and the surrounding environment, has developed complex protective responses against environmental insults, such as xenobiotic and ultraviolet (UV) radiation. However, the UVB, which have a wavelength of 280-320 nm, can penetrate the epidermis, the outermost layer of skin, and excessive exposure results in DNA damage and a cutaneous inflammatory reaction commonly known as sunburn, which increases skin cancer risks. Although many of the factors and mechanisms that instigate UVB-induced inflammatory responses have been well characterized, the intracellular machinery through which epidermal homeostasis is restored after UVB radiation remains unclear. Previously, we and others have shown that NLRP3 (NLR family pyrin domain containing 3) inflammasome in human epidermal keratinocytes participate in UVB-induced inflammatory responses. Inflammasomes are cytoplasmic protein complexes that recognize a broad range of foreign pathogens, host-derived danger signals, and environmental stimuli, and facilitate the release of IL-1β (interleukin-1β) and IL-18 to instigate inflammatory responses. Although inflammasomes are critical for defense against pathogens, their aberrant regulation, particularly in response to host-derived danger signals and metabolites, can cause inappropriate sterile inflammatory responses, which occur in the absence of pathogens and form the basis of many chronic and degenerative diseases. Inflammatory epithelial diseases are spurred by the concomitant dysregulation of immune and epithelial cells.

In this context, we aimed to explore the intracellular machinery that serves to limit UVB-induced NLRP3 inflammasome activation and block excessive inflammatory responses in human epidermal keratinocytes [1]. We found that pharmacological inhibition of autophagy with 3-methyladenine, SAR405 or SBI-0206965, promoted UVB-induced NLRP3 inflammasome activation in keratinocytes. Unexpectedly, however, small interfering RNA-mediated gene silencing of ATG5 or ATG7, which are thought to be critical for conventional autophagy, had no effect, whereas gene silencing of Beclin1, which is essential not only for conventional autophagy but also for ATG5/ATG7-independent alternative autophagy, promoted UVB-induced inflammasome activation in keratinocytes. We further found that the gene silencing of either RAB9 or WIPI3, which are essential for ATG5/ATG7-independent alternative autophagy, but not for ATG5/ATG7-dependent conventional autophagy, also promoted UVB-induced inflammasome activation in keratinocytes. These results indicate that ATG5/ATG7-independent alternative autophagy preferentially participates in the suppression of UVB-induced inflammasome activation in human keratinocytes. The mammalian macroautophagy (generally referred to as autophagy) system employs several different machineries to maintain cellular homeostasis, such as ATG5/ATG7-dependent conventional autophagy and ATG5/ATG7-independent alternative autophagy (also called Golgi membrane-associated degradation [GOMED]), in a stimulus-dependent and context-dependent manner. Therefore, to investigate whether ATG5/ATG7-independent alternative autophagy actually operates in UVB-exposed keratinocytes, we evaluated the cellular level of Ulk1 phosphorylation at Ser^746^, which is a marker of ATG5/ATG7-independent alternative autophagy, in UVB-exposed keratinocytes. As expected, the p-Ulk1^746^ signal was significantly increased and emerged in the Golgi upon UVB radiation. ATG5/ATG7-independent alternative autophagy is known to be induced by certain genotoxic stresses, such as exposure to DNA-damaging chemotherapeutic agents, and UVB in sunlight causes a genotoxic stress through its direct and indirect DNA-damaging effects. UVB-induced alternative autophagy in keratinocytes may be triggered at least in part by DNA damage.

We hypothesized that ATG5/ATG7-independent alternative autophagy eliminates undesirable intracellular stimuli that are recognized by an intracellular sensor protein, NLRP3. This idea was inspired by the increasing evidence suggests that ATG5/ATG7-independent alternative autophagy predominantly participates in the removal of damaged mitochondria during dynamic metabolic reprogramming associated with maturation of erythrocytes, reprogramming of induced pluripotent stem (iPS) cells, high fat diet-induced cardiomyopathy, and DNA-damaging chemotherapy drug treatment. In addition, damaged mitochondria trigger NLRP3 inflammasome activation. In order to test whether there was a correlation between UVB radiation, NLRP3 and damages mitochondria, we evaluated whether damaged mitochondria are generated by UVB radiation. We found that damaged mitochondria were highly accumulated in UVB-irradiated keratinocytes when ATG5/ATG7-independent alternative autophagy, but not conventional autophagy, was inhibited. Consistent with this result, NLRP3 was co-localized with the mitochondrial outer membrane protein TOM20 in UVB-exposed keratinocytes under conditions of inhibition of ATG5/ATG7-independent alternative autophagy, but not conventional autophagy. These results suggest that UVB-induced damaged mitochondria are recognized by NLRP3, leading to activation of NLRP3 inflammasomes in keratinocytes, while alternative autophagy, rather than conventional autophagy, mediates the clearance of damaged mitochondria to block excessive NLRP3 inflammasome activation (Figure 1).

**Figure 1.**
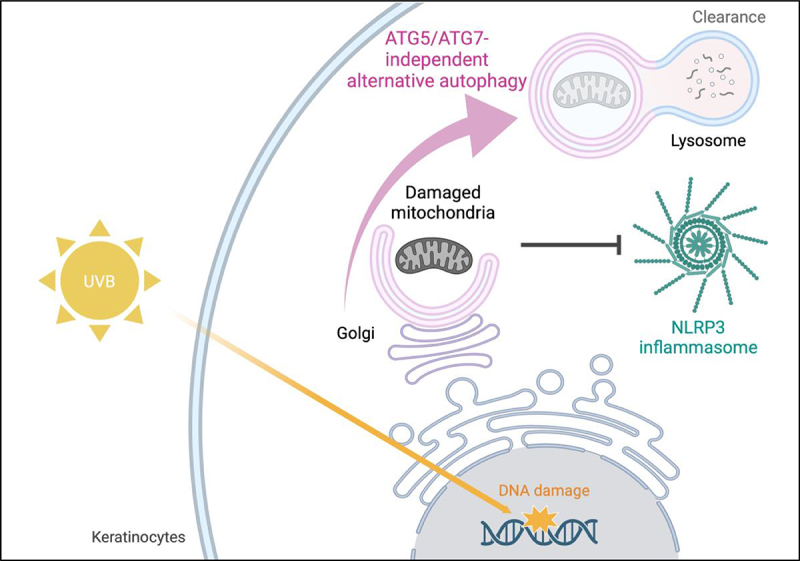
The UVB-induced ATG5/ATG7-independent alternative autophagy eliminates damaged mitochondria, which trigger NLRP3 inflammasome activation in human keratinocytes. Created with BioRender.com

Mitochondria have multiple functions, including energy generation and metabolism regulation, and play an important role as a hub for innate immune responses, particularly NLRP3 inflammasome and retinoic acid-inducible gene I (RIG-I)-like receptors (RLRs)-mediated signaling pathways. Healthy mitochondria are subjected to quality control processes, including clearance of damaged mitochondria through a selective form of autophagy, known as mitophagy, to fine-tune their status in response to physiological adaptations, as well as intrinsic and extrinsic stresses. Although the mechanism of ATG5/ATG7-dependent conventional mitophagy is relatively clear at the molecular level, the mechanism of ATG5/ATG7-independent alternative autophagy-mediated mitochondrial clearance remains to be elucidated. For instance, SQSTM1/p62 (sequestosome 1) is an important selective autophagy receptor during certain types of conventional mitophagy, but not during ATG5/ATG7-independent alternative mitophagy. To elucidate the precise pathway of ATG5/ATG7-independent alternative mitophagy, identification of the specific selective autophagy receptor for damaged mitochondria is required.

In conclusion, our findings indicate a protective role of ATG5/ATG7-independent alternative autophagy against sunburn-related inflammatory responses in human keratinocytes through the clearance of damaged mitochondria, uncovering the intracellular machinery that restores epidermal homeostasis after UV radiation. ATG5/ATG7-independent alternative autophagy might be a new therapeutic target to treat sunburn and prevent the related skin cancers.
